# Crossing the Rift valley: using complete mitogenomes to infer the diversification and biogeographic history of ethiopian highlands *Ptychadena* (anura: Ptychadenidae)

**DOI:** 10.3389/fgene.2023.1215715

**Published:** 2023-08-03

**Authors:** M. L. Lyra, S. Kirchhof, S. Goutte, A. Kassie, S. Boissinot

**Affiliations:** ^1^ New York University Abu Dhabi, Abu Dhabi, United Arab Emirates; ^2^ Animal Biodiversity Directorate, Ethiopian Biodiversity Institute, Addis Ababa, Ethiopia; ^3^ Department of Zoological Sciences, College of Natural and Computational Sciences, Addis Ababa University, Addis Ababa, Ethiopia; ^4^ Center for Genomics and Systems Biology, New York University Abu Dhabi, Abu Dhabi, United Arab Emirates

**Keywords:** mitogenomes, low-coverage whole genome sequencing, phylogenomics, population structure, ancestral range, geology, climatic oscillation

## Abstract

The Ethiopian Highlands are considered a biodiversity hotspot, harboring a high number of endemic species. Some of the endemic species probably diversified *in situ*; this is, for example, the case of a monophyletic clade containing 12 known species of grass frogs of the genus *Ptychadena*. The different species occur at elevations ranging from 1,500 to above 3,400 m and constitute excellent models to study the process of diversification in the highlands as well as adaptations to high elevations. In this study, we sampled 294 specimens across the distribution of this clade and used complete mitogenomes and genome-wide SNP data to better understand how landscape features influenced the population structure and dispersal of these grass frogs across time and space. Using phylogenetic inference, population structure analyses, and biogeographic reconstructions, we found that the species complex probably first diversified on the south-east side of the Great Rift Valley. Later on, species dispersed to the north-west side, where more recent diversification occurred. We further demonstrate that *Ptychadena* species have dispersed across the Great Rift Valley at different times. Our analyses allowed for a more complete understanding of the contribution of geological events, biogeographic barriers and climatic changes as drivers of species diversification and adaptation in this important biogeographic region.

## 1 Introduction

The Ethiopian Highlands constitute one of the major centers of endemism and biodiversity in continental Africa (Williams et al., 2005; Fashing et al., 2022). Beginning as a single land massif with a complex geological history of uplift and volcanism (Yemane et al., 1985; Wolfenden et al., 2004; Bonini et al., 2005; Corti, 2009; Sembroni et al., 2016; Xue et al., 2018), this region was split by the development of the Great Rift Valley (GRV) during the late Miocene and Pleistocene, creating an impressive gradient of elevations and climates, high environmental heterogeneity and diverse vegetation types.

The GRV crosses the Ethiopian Highlands along a north-east to south-west axis, dividing the highlands into two major blocks: the south-east massif, adjacent to the arid zone of the Horn of Africa, and the north-west massif, the largest contiguous area of high elevations in Africa. The north-west massif is further divided by the Blue Nile river valley (known as the Abay river in Ethiopia) that separates the Gojjam highlands from the Wollo highlands and Simien mountains of the north-west (Gani et al., 2007). The south-east massif is incised by the Shebelle and Dawa-Genale river systems, which originate from the Ahmar and Bale mountains, respectively (Xue et al., 2018).

The bottom of the GRV is significantly drier in comparison to the more humid rift flanks and the cooler plateaus, and is currently a significant barrier to dispersal for several taxa that inhabit the highlands, including mammals (Gottelli et al., 2004; Bryja et al., 2018; Komarova et al., 2021; Razgour et al., 2021), birds (Manthey et al., 2022), anurans (Evans et al., 2011; Freilich et al., 2016; Manthey et al., 2017; Reyes-Velasco et al., 2018a; Reyes-Velasco et al., 2018b), reptiles (Largen and Spawls, 2010; Ceccarelli et al., 2014) and plants (Kebede et al., 2007; Kidane et al., 2022). Some of these studies also show that the hydrological systems, such as the Blue Nile (Abay) valley, have played an important role as geographical barriers for dispersion of some of the Ethiopian Highlands taxa.

Despite being a hotspot for biodiversity, the Ethiopian Highlands have been identified as one of the African regions with the highest proportion of potentially threatened species (Stévart et al., 2019; Fashing et al., 2022). This is mainly because the region is under critical threat due to rapid human population growth and expansion of agriculture (Stephens et al., 2001; Gower et al., 2013; Stévart et al., 2019; Fashing et al., 2022). In addition, contemporary climatic changes also negatively affect the montane ecosystems (Asefa et al., 2020).

Amphibians are the most endangered group of vertebrates on Earth, with more than 40% of species evaluated as threatened or extinct (IUCN, 2023). In Ethiopia, 79 species of amphibians are currently known (25% described in the last 2 decades) and 39 of those are endemic to the country (Largen, 2001; Amphibiaweb, 2023; Frost, 2023). More than 90% of the endemic amphibians are known only from the Ethiopian Highlands. From those, at least 30% are listed under threatened categories at the IUCN red list and ∼50% are listed as “Data Deficient” or have not yet been assessed (Amphibiaweb, 2023; IUCN, 2023). The *Ptychadena* species of the *P. neumanni* species complex (*sensu* Freilich et al., 2014; Ethiopian Highlands *Ptychadena* herein) form a monophyletic clade containing 12 species that probably diversified *in situ* (Goutte et al., 2021). Previous work, based on an incomplete taxonomic resolution, suggested that most of the species diversified in allopatry and that the GRV played an important role in this process (Freilich et al., 2014; Smith et al., 2017a; Reyes-Velasco et al., 2018a). In addition, it was found that some species present different levels of population structure that are best explained by the topography of the highlands (Freilich et al., 2016; Reyes-Velasco et al., 2018a). However, until 2014, only five species of the *P. neumanni* species complex were named, and these were described using mainly morphological data. Since then, the taxonomy has been largely resolved and seven new species were described using a combination of morphological analyses, bioacoustics data and molecular analyses (Smith et al., 2017a; Goutte et al., 2021; Reyes-Velasco et al., 2021).

Species belonging to the Ethiopian Highlands *Ptychadena* are present on both sides of the GRV and have colonized different elevations, ranging from 1,500 to above 3,400 m (Largen and Spawls, 2010; Freilich et al., 2014; Reyes-Velasco et al., 2018a), and a variety of habitats, including pristine vegetation such as forests and grasslands as well as meadows and cultivated fields. The current distribution of the species in this clade and the adaptation of *Ptychadena* species to different elevations and environments, make this group an excellent model to study diversification in the Ethiopian Highlands.

Here we aim to reconstruct the phylogeography of the monophyletic clade of Ethiopian Highlands *Ptychadena* taxa and to understand the geographic ordination of genotypes to uncover potential processes underlying diversification in this topographically diverse environment. We use phylogenetic inference, population structure analyses, and biogeographic reconstructions, to determine the timing and patterns of diversification of Ethiopian Highlands *Ptychadena*. In the future, comparative analyses of underlying diversification processes across different organism groups might broaden our understanding of the role of topography and climate in the evolution of the Ethiopian Highlands biodiversity, and in particular in the evolution of endemic species. This knowledge is the fundamental basis for improving conservation strategies for this global hotspot of biodiversity.

## 2 Material and methods

### 2.1 Sampling strategy and sequencing

**FIGURE 1:**
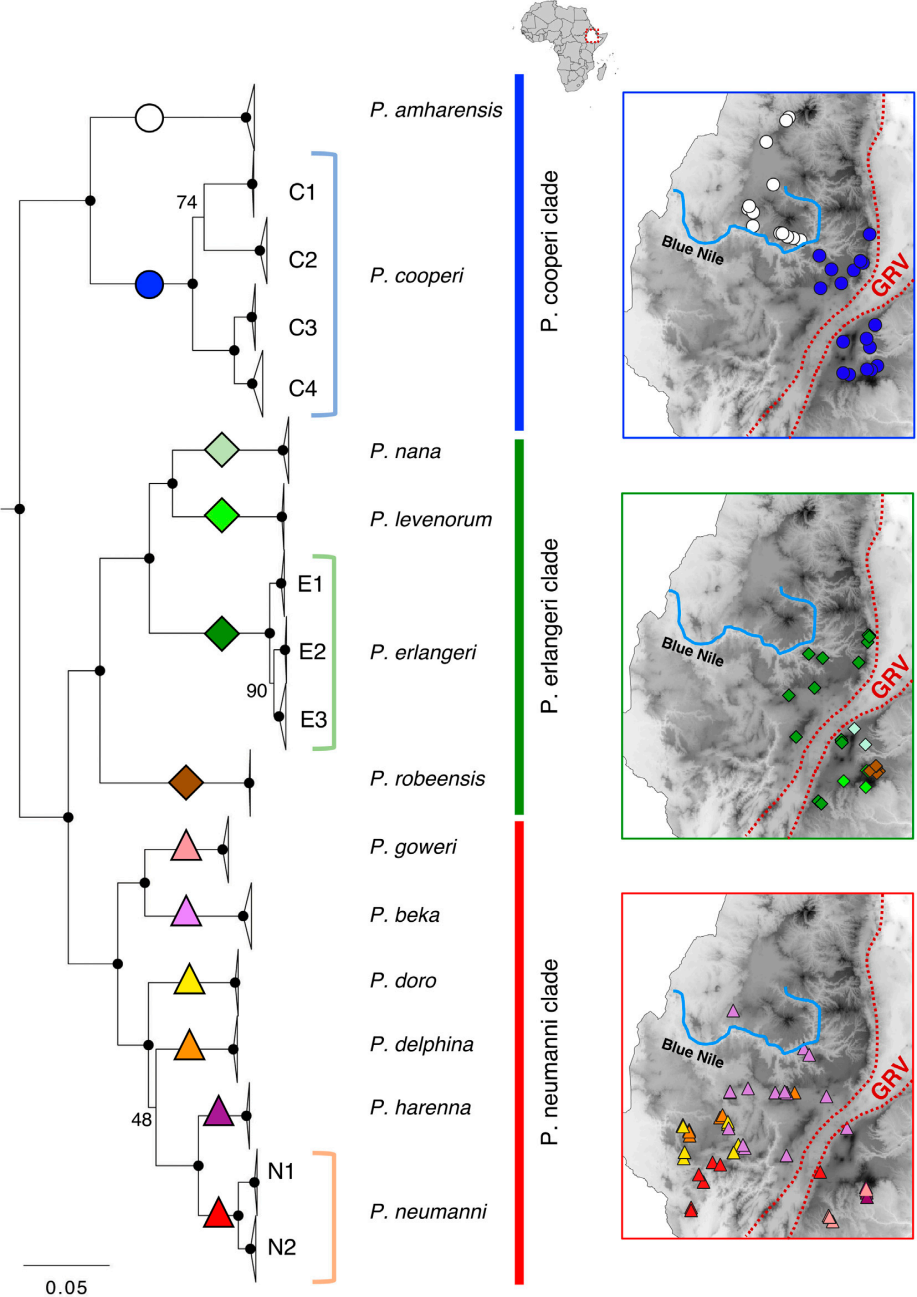
Maximum-likelihood mitogenomic tree and geographic distribution of Ethiopian Highlands *Ptychadena.* Nodes with high bootstrap support (BS = 100) are noted with black circles. Nodes with lower support are indicated on the figure. Colored symbols highlighted in the tree represent species, and the respective distributions are shown in the maps following the same color and symbol scheme. GRV = Great Rift Valley. Blue Nile = Blue Nile (Abay) river.

We sampled 294 individuals across all 12 species of the monophyletic Ethiopian Highlands *Ptychadena*, spanning the known geographical range of each species (Figure 1; Table 1; specimens’ information is provided in Supplementary Table S1). The geographical range for each species had previously been described by Goutte et al. (2021) based on our own collection efforts covering more than 520 different Ethiopian localities, as well as observations by other authors (Mengistu, 2012; Smith et al., 2017a). We selected from the maps of Goutte et al. (2021) localities representative of range of each species. The samples used in the present study were collected between 2011 and 2019, and several of them were included in previously published works (Freilich et al., 2014; Freilich et al., 2016; Reyes-Velasco et al., 2018a; Goutte et al., 2021; Goutte et al., 2022). Our study was approved by the Institutional Animal Care and Use Committee at Queens College (IACUC; Animal Welfare Assurance Number A32721-01) and at New York University School of Medicine (IACUC; Protocols 16-0002, 19-0001 and 19-0003). Specimens were sampled according to permits provided by the Ethiopian Wildlife Conservation Authority (DA31/305/05, DA5/442/13, DA31/454/07, DA31/192/2010, DA31/230/2010, DA31/7/2011, and DA31/02/11). Voucher specimens are deposited at the Zoology Museum of the University of Addis Ababa, Ethiopia and tissue samples are deposited at the Vertebrate Tissue Collection at New York University Abu Dhabi (NYUAD). The geographical coordinates of the sampling points were obtained with Garmin GPS, model Oregon 600.

**TABLE 1:** Number of specimens included in the mitogenome analysis (N mtDNA) and nuclear SNPs (N nuclear), summary statistics of mitochondrial nucleotide diversity (π) and Tajima’s D test (*p < 0.05; in bold) and summary of SNP dataset used in analyses (N SNPs).

Species clade	Species	N mtDNA	π (%)	Tajima’s D	N nuclear	N SNPs
*P. cooperi*	*Ptychadena amharensis*	33	0.32	−2.7145*	15	1,014,633
	*Ptychadena cooperi*	57	3.47	1.3141	27	1,254,730
	Clade C1 - Guru	5	0.08	−1.0529	5	
	Clade C2 - Bale	20	0.11	−2.0634*	5	
	Clade C3 - Arsi	6	0.19	−0.5038	5	
	Clade C4 - West	26	0.38	0.3126	12	
*P. erlangeri*	*Ptychadena erlangeri*	25	0.64	−1.1718	16	596,677
	Clade E1 - Kibre	2	0.3	NA	2	
	Clade E2 - Assela	4	0.1	−1.3421	4	
	Clade E3 - West	19	0.24	−1.7391	10	
	*Ptychadena nana*	9	0.16	−2.2283*	3	3,322,166
	*Ptychadena levenorum*	10	0.12	−1.5155	9	2,996,933
	*Ptychadena robeensis*	61	0.03	−2.7251*	10	1,286,041
*P. neumanni*	*Ptychadena neumanni*	11	0.99	0.8315	8	1,109,937
	Clade N1 - West-East	4	0.18	−2.0833*	4	
	Clade N2 - West	7	0.3	−0.5823	4	
	*Ptychadena harenna*	6	0.13	−1.6637	6	1,457,962
	*Ptychadena delphina*	15	0.24	−0.8836	9	4,334,002
	*Ptychadena doro*	12	0.18	−0.9875	7	2,135,708
	*Ptychadena beka*	44	0.27	−1.7880	16	886,444
	*Ptychadena goweri*	11	0.25	−1.0901	9	1,517,507
Total		294			135	27,709

For the phylogenetic inference, we also included three additional species of *Ptychadena* (*Ptychadena anchietae* (Bocage, 1868), *Ptychadena nuerensi*s Smith et al., 2017b, and *Ptychadena wadei* Largen, 2001), 5 taxa representing other ranoidea and one sample of *Sooglossus thomasseti* (Boulenger, 1909) (Sooglossidae) to root the tree, following Feng et al., 2017 (Supplementary Table S1).

The total genomic DNA was extracted from tissues (muscle or liver) preserved in 95% ethanol or RNAlater (Invitrogen) using DNeasy blood and tissue kit (Qiagen, Valencia, CA) and following manufacturers’ protocol. Samples were quantified with high sensitivity kit in a Qubit fluorometer (Life Technologies). Illuminia TRU-seq paired end libraries were generated using 80 ng of DNA per sample and sequenced to approximately 2x coverage using Illumina Nextseq 500/550 or HiSeq2500 (100 bp paired-end reads) at the Sequencing Core Facility of New York University Abu Dhabi, United Arab Emirates.

### 2.2 Mitogenome assembly and SNP calling

To assemble the mitochondrial genome (mitogenome) of each sampled individual, we first removed low quality bases and sequencing adapter contamination from raw data using *Trimmomatic* (V.0.39; Bolger et al., 2014). Then we subsampled 10 million read pairs of the trimmed reads using *Seqtk* (V.1.3; Li, 2018), and used *GetOrganelle* (V.1.7.5; Jin et al., 2020) to *de novo* assemble the mitogenomes, under default settings using all subsampled reads. When analyses resulted in alternative assemblies, we checked the output graph using *Bandage* (V.0.9; Wick et al., 2015), and used only the consensus sequence between all assemblies for analyses (see results for details). The final mitogenome annotation was carried out using *MITOS* (V.2; Donath et al., 2019) and *tRNAscan* (Chan and Lowe, 2019). The protein-coding regions were translated in *Geneious* prime 2022.1.1 (https://www.geneious.com) to confirm that no indels or stop codons were present.

To assess concordance between mitochondrial and nuclear genetic structure we obtained single nucleotide polymorphisms (SNPs) on a subset of samples, totalizing 135 individuals (approximately one individual per species per locality). For calling the SNPs, we first aligned quality trimmed reads of each individual to a chromosome-scale *Ptychadena robeensis* Goutte, Reyes-Velasco, Freilich, Kassie and Boissinot, 2021 reference genome (in prep), using the *BWA-mem* short read alignment approach (V.0.7.17; Li and Durbin, 2009). We then used *Samtools* (V.1.9; Danecek et al., 2021) and Picard (V.2.21.1, http://broadinstitute.github.io/picard) to convert and clean files. Variant calling was performed with *GATK* HaplotypeCaller (V.3.8; Poplin et al., 2017) and the resulting GVCFs were combined and genotyped for each species, using *GATK* CombineGVCFs and *GATK* GenotypeGVCFs, respectively (McKenna et al., 2010). Filtering was performed in VCFtools (V.0.1.16; Danecek et al., 2011), with minor-allele frequency (MAF) threshold of 5%, allowing 1% of missing data, minimum quality score of 30 and removing all indels.

The mitogenome sequences were submitted to GenBank (accession numbers provided in Supplementary Table S1). The raw reads are accessible through the National Center for Biotechnology Information (NCBI) at the Sequence Read Archive (SRA) associated with BioProject ID PRJNA975347. The computational pipelines used for the analyses described above are available at https://github.com/marilyra/Ptychadena_biogeography.

### 2.3 Phylogenetic inference and population structure

To reconstruct the phylogenetic and phylogeographic history of the 12 species of Ethiopian Highlands *Ptychadena*, we first extracted the sequences of the two rRNAS (12S and 16S) and the 13 protein-coding genes (CDS), using Geneious Prime^®^ 2022.1.1 (https://www.geneious.com). We aligned each gene independently using MAFFT 7 (Katoh and Standley, 2013), under the strategies E-INS-i for rRNA genes and G-INS-i for CDSs, and concatenated the independent alignments in Geneious. The final alignment consisted of 303 samples and was 13,937 bp long. We predefined two partitions: one for the combined 12S and 16S rRNAs, and one for each codon position (first, second, and third) of the protein-coding genes. Then, we conducted a maximum likelihood analysis (ML) on the total mitogenomic matrix using RAxML 8.2.10 (Stamatakis, 2014), with 100 independent searches for the best tree and 1000 non-parametric bootstrap replicates, and the GTRCAT approximation, considering the four predefined partitions.

The mitochondrial nucleotide diversity (Nei, 1987), haplotype diversity (Nei and Tajima, 1981) and Tajima’s D (Tajima, 1989) were calculated for each species and each population using *Pegas* package (Paradis, 2010) in R software (R Core Team, 2022). The population structure of the nuclear genome was investigated using principal component analysis (PCA) based on the variance-standardized relationship matrix computed using *plink* (V.1.9; Purcell et al., 2007), and plotted using *ggplot2* package (Wickham, 2016) in R software.

We used QGIS software V.3.26.1 (http://www.qgis.org) to create spatial distribution maps of species and genetically structured populations within species according to clades recovered in the phylogenetic tree.

### 2.4 Time-calibrated tree

To estimate the timing of diversification events in the Ethiopian Highlands *Ptychadena*, we reconstructed a time-calibrated mitochondrial tree using only one individual per species and per clade, selected based on the ML tree. The three additional species of *Ptychadena* were included and we used *Phrynobatrachus keniensis* Barbour and Loveridge, 1928, to root this tree (Supplementary Table S1). The two rRNAs and all CDS were extracted, genes were aligned and concatenated as described above. We used the same pre-defined partition and the model of evolution for each partition was selected using jModelTest2 (Darriba et al., 2012), according to the Bayesian Information Criterion (BIC).

The timings of divergence were estimated using BEAST v2.7.1 (Bouckaert et al., 2019) under a birth-death tree prior. We parameterised unlinked substitution models, using GTR + I + G for each partition, as estimated by jModelTest2, and time calibration was implemented using an uncorrelated relaxed lognormal clock model of the distribution of rates among branches for each partition. The Markov chain Monte Carlo (MCMC) parameters were set to four independent runs of 20 million iterations each, sampling every 2000 iterations and discarding the first 25% iterations as burn-in. We assessed stationarity, convergence between runs, and effective sample sizes (>200) with Tracer (v.1.7, Rambaut et al., 2018), we combined the log files of the independent runs using LogCombiner (v.2.7, Bouckaert et al., 2019), and we extracted the Maximum Clade Credibility (MCC) tree in TreeAnnotator (V.2.7, Bouckaert et al., 2019).

To calibrate the tree, we used a fossil of the Ptychadenidae family described by Blackburn et al. (2015) from the Oligocene Nsungwe formation of Tanzania, dated to be between 25.5 and 24.5 Mya old. Specifically, we used a log normal distribution prior, with a minimum age of 25 Mya, standard deviation of 1.2, and 95% CI from 25.2 Mya to 54.6 Mya. The maximum age was set considering the split between the families Ptychadenidae and Phrynobatrachidae at 54.5 Mya (48.3–60.1 Mya) following Feng et al. (2017).

### 2.5 Biogeographical analysis

For the inference of ancestral areas we used the time-calibrated phylogeny of the Ethiopian Highlands *Ptychadena*, excluding outgroups, and used the BioGeoBEARS package (Matzke, 2013) implemented in RASP V.4 (Reconstruct Ancestral State in Phylogenies; Yu et al., 2020).

Based on the present distribution patterns, each species and population was assigned to the following biogeographical areas: (E) East of the GRV; (W) West of the GRV; and (N) North of the Blue Nile valley (see Figure 3). We compared three potential models: 1) the Dispersal Extinction Cladogenesis model (DEC; Ree and Smith, 2008); 2) a likelihood version of the Dispersal-Vicariance model (DIVALIKE; Ronquist, 1997; Matzke, 2013), and 3) a likelihood version of the BayArea (BBM) model (Landis et al., 2013). We also compared versions of these models allowing “jump dispersal”, to determine the influence of founder-event dispersal on biogeographic patterns. To account for phylogenetic uncertainty, 100 random post-burn-in trees yielded from BEAST2 for the above divergence time estimation were included, with removal of outgroup taxa. We ran the BioGeoBEARS biogeographical stochastic mapping 100 times to determine biogeographical event counts for the best-fit model (Dupin et al., 2017). For our data the DIVALIKE + J was selected based on the best-fitting model, according Akaike Information Criterion (AICc, wt value; Supplementary Table S2), and this was subsequently used to infer the most likely biogeographic history of species.

## 3 Results

### 3.1 Mitochondrial genomes

The gene order arrangement and gene content of the mitogenomes for all species of the Ethiopian Highlands *Ptychadena* are similar to most other Neobatrachia (Zhang et al., 2013). All assembled mitogenomes contain the two ribosomal genes (12S and 16S rRNA), the 13 protein-coding genes, 22 tRNAs and a major non-coding region (CR), in between Cytochrome B (CytB) and the tRNA cluster Leucine-Threonine-Proline-Phenylalanine (LTPF cluster). In the *P. amharensis* Smith et al., 2017b and *P. cooperi* (Parker, 1930) mitogenomes we found a relatively large non-coding region between tRNA-Leucine and tRNA-Threonine (>200 bp). For all species we also found a non-coding region (∼70 bp) between the tRNA Histidine-Serine cluster and the gene NADH-5. We could not completely assemble the CR region for any species due to the presence of many sequence repeats, which could not be resolved with confidence using the short-read sequencing data.

### 3.2 Phylogenetic inference and population structure

We recovered three well-supported clades for the Ethiopian Highlands *Ptychadena* as previously described (Freilich et al., 2014; Reyes-Velasco et al., 2018a; Goutte et al., 2021): The *P. cooperi* clade (containing *P. amharensis* and *P. cooperi*), the *P. erlangeri* clade (*P. erlangeri* (Ahl, 1924), *P. levenorum* Smith et al., 2017b *, P. nana* Perret, 1980, and *P. robeensis*) and the *P. neumanni* clade [*P. beka* Goutte, Reyes-Velasco, Freilich, Kassie and Boissinot, 2021, *P. delphina* Goutte, Reyes-Velasco, Freilich, Kassie and Boissinot, 2021 *, P. doro* Goutte, Reyes-Velasco, Freilich, Kassie and Boissinot, 2021, *P. goweri* Smith et al., 2017b, *P. harenna* Largen, 2001, and *P. neumanni* (Ahl, 1924)]. The *P. cooperi* clade was recovered as sister to the *P. neumanni* + *P. erlangeri* clade (Figure 1). The topology of the ML tree with all samples and the Bayesian time-calibrated tree (Figure 3; Supplementary Figure S1) were largely congruent and strongly supported all the species relationships (bootstrap support, BS = 100; and posterior probability, PP = 1), except for the position of *P. delphina.* The ML tree recovered *P. delphina* as sister to *P. harenna* + *P. neumanni* (Figure 1), with a BS = 48 and the time-calibrated tree recovered *P. delphina* as sister to *P. doro*, and these two as sisters to *P. harenna* + *P. neumanni* (PP = 1; Supplementary Figure S1).

All clades contain species present on both sides of the GRV (Figure 1). In the *P. cooperi* clade, *P. amharensis* is present only on the western side north of the Blue Nile valley, while *P. cooperi* is present both on the eastern and western sides of the GRV (but only south of the Blue Nile valley). In the P. *erlangeri* clade, all species are present on the eastern side of the GRV, and only *P. erlangeri* occurs also west of the GRV. The distribution ranges of species belonging to the *P. neumanni* clade is more complex. Two species (*P. goweri* and *P. harenna*) are present only on the eastern side of the GRV, two species (*P. doro* and *P. delphina*) are present only on the western side of the GRV, and two species are present on both sides, *P. neumanni* and *P. beka.* Interestingly, *P. beka* is also present north of the Blue Nile valley. While species from the three clades are present on both sides of the GRV, the species from the *P. neumanni* clade apparently do not occupy the elevation above 2,700 m as the species from the other two clades (Figure 3; Supplementary Table S1).

**FIGURE 2:**
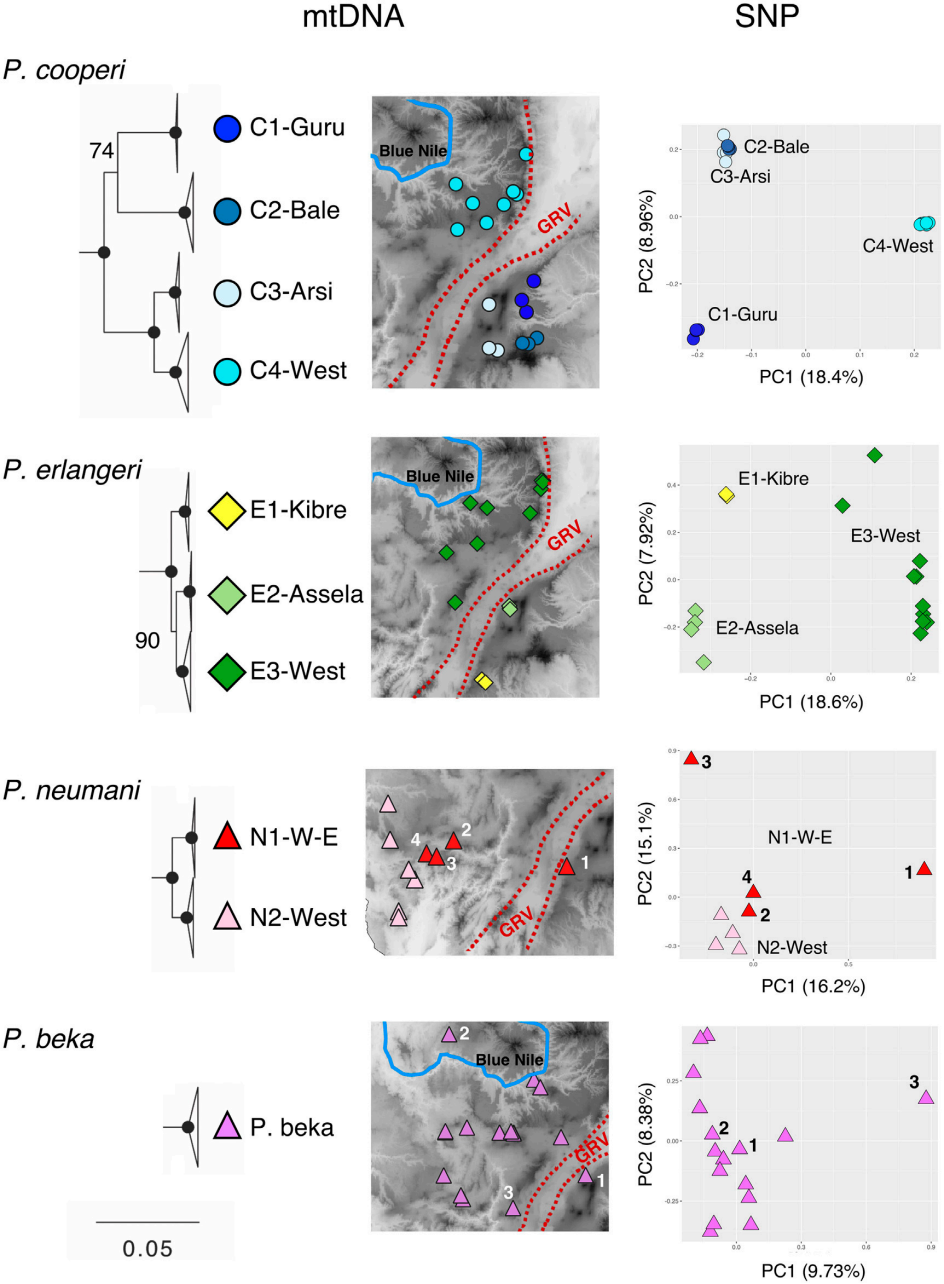
Genetic structure in Ethiopian Highlands *Ptychadena*. Colored symbols in maps represent structured populations identified in the mitogenomic dataset. PCA plots on the right side were obtained from the nuclear SNPs of each species and colored according to the mitochondrial clades. From top to bottom: *Ptychadena cooperi*, *P. erlangeri*, *P. neumanni* and *P. beka*. For P. *neumanni* we identified with numbers on map and PCA the individual from clade N1-W-E included on analyses. For P. *beka* we identified with numbers individuals found on West of Great Rift Valley (GRV) and North of Blue Nile valley; also the locality of the most divergent individual found in SNP data.

We found population structure for only three of the 12 species analyzed (Figure 2). Using the mitogenomic data we found four structured populations within *P. cooperi*: three populations are geographically distributed in different mountain regions in the east of the GRV (C1-Guru, C2-Bale and C3-Arsi) while the fourth population (C4-west) is present west of the GRV (Figure 2). The nuclear data partially support the mitogenomic structure. The PCA analysis, including ∼1.25 million SNPs (Table 1), recovered only three *P. cooperi* populations, one in the west and two in the east, while C2-Bale and C3-Arsi are not differentiated (Figure 2). For *P. erlangeri*, we found three populations, one in the west and two in the east of the GRV. Both mitochondrial and nuclear data (0.6 million SNPs) support this finding. For *P. neumanni*, we found two clades with mitochondrial data, but no geographic structure for the nuclear data (PCA included ∼1.1 million SNPs). The two mitochondrial clades are present on the western side of the GRV, and we could not find any obvious geographical barrier between them. However, it is important to note that we analyzed only 4 individuals per clade using nuclear data for this species, which might be insufficient to show proper geographic patterns.

We also explored the nuclear population structure of *P. beka*, since it is the only species present in all three geographical areas of the Ethiopian Highlands detected by us. The PCA analysis (0.9 million SNPs) supports the mitochondrial findings, which showed no geographical structure; the population found north of the Blue Nile valley and the one east of the GRV being nested within the diversity found in the highlands west of the GRV and south of the Blue Nile (Figure 2). For the other species, neither mitochondrial (Figure 1) nor nuclear data presented population structure (data not shown).

In general, we found very high haplotype diversity for the mitochondrial data (within 0.98–1 in all populations; 269 different mitochondrial haplotypes for 294 individuals), and relatively low nucleotide diversity (from 0.08% to 3.47%). The highest nucleotide diversities were found in the species with population structure (*P. cooperi, P. erlangeri* and *P. neumanni*). The values for Tajima’s D suggested that *P. amharensis, P. nana, P. robeensis, P. neumanni*-N1*, P. cooperi*-C2-Bale are not evolving in mutation–drift equilibrium and constant population size (significative negative Tajima’s D values; Table 1).

Based on our data, species that are currently restricted to the eastern side of the GRV (*P. levenorum, P. nana, P. robeensis, P. goweri* and *P. harenna*) have smaller distribution ranges than the ones present in the west, or on both sides (Figure 1). We also found some species in sympatry (Figure 1; Supplementary Table S1): *P. cooper*i with *P. erlangeri* and *P. levenorum*; *P. levenorum* and *P. robeensis*; *P. goweri* and *P. harenna*; *P. doro* and *P. delphina*; and *P. neumanni* and *P. doro*.

### 3.3 Biogeographic analyses and times of divergence

**FIGURE 3:**
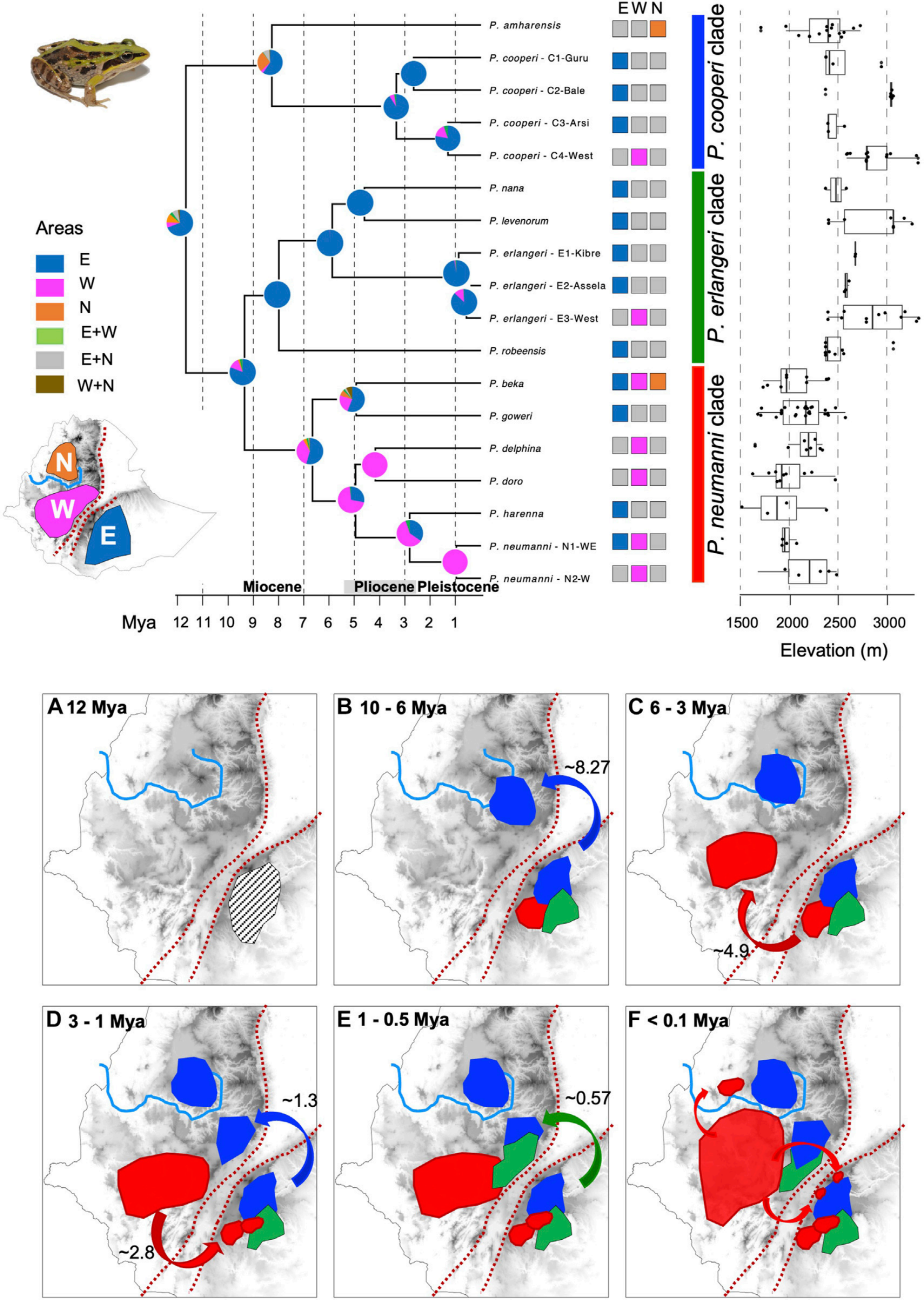
Time calibrated phylogeny based on mitogenomes and ancestral area reconstruction for the diversification of the Ethiopian Highlands *Ptychadena.* The three biogeographical areas of diversification (East of the Great Rift Valley (GRV), West of GRV and North of Blue Nile valley) are shown on the map left of the tree. Species and population distribution are coded near the terminals of the tree. We also depicted the elevation range for each species (on the right). Circles in the nodes show probabilities of ancestral occurrence according to the three biogeographical areas. Panels (a) to (f) shows the hypothetical distribution of each species clade (*P. cooperi* clade in blue, *P. erlangeri* clade in green and *P. neumanni* clade in red) and the events of dispersal through time. Only the most probable scenarios (probability score >50%) are shown in the panels.

The crown age of the most recent common ancestor for Ethiopian Highlands *Ptychadena* was estimated to be around 11.7 Mya (15.494–8.827 Mya; middle-late Miocene) and the most probable ancestral area for the ancestor of the entire group was the eastern side of the GRV (probability = 69%; Figure 3, panel a; Supplementary Table S2). Analyses also suggest that the species diversification within clades and the subsequent dispersion to the north-western side of the GRV was asynchronous (Figure 3, panel b-f).

In the *P. cooperi* clade, the ancestor of the *P. amharensis* lineage most likely dispersed from the east across the GRV and diverged from the *P. cooperi* lineage around 8.27 Mya (Figure 3; panel b). Later *P. amharensis* expanded its range to north of the Blue Nile valley, the current distribution of the species. In turn, *P. cooperi* diversified in the east into three genetic clusters and only dispersed to the west in the middle Pleistocene (C4-west; ∼1.3 Mya; Figure 3, panel d). The species from the *P. erlangeri* clade diversified on the east side of the GRV between 8 Mya and 4.6 Mya. Only *P. erlangeri* dispersed to the west at around ∼0.57 Mya, resulting in the E3-west genetic cluster (Figure 3, panel e). The diversification process of the *P. neumanni* clade across both sides of the GRV was ambiguous in our analyses. The common ancestor for the clade was either in the east (probability = 54%) or in the west of the GRV (probability = 37%). In the scenario where the ancestral species occurred in the east, both *P. beka* and the ancestor of the clade containing *P. doro*, *P. delphina*, *P*. *harenna* and *P. neumanni*, would have dispersed to the west around 4.9 Mya (Figure 3, panel c). Then, *P*. *harenna* would have dispersed back to the east at the end of the Pleistocene (∼2.8 Mya; Figure 3, panel d). In the scenario where the ancestor occurred in the west, it would have dispersed from east to west between ∼9.3 Mya and ∼6.6 Mya. Then, *P. goweri* would have dispersed to the east at around 4.9 Mya, followed by *P. harenna* at ∼2.8 Mya. In both scenarios, *P. beka* most probably dispersed to the east of the GRV and the north of the Blue Nile valley in very recent time since these populations are not genetically different (Figure 3, panel f). The same pattern is likely for *P. neumanni*, which dispersed very recently to the east (Figure 3, panel f). Age information for all nodes, the 95% CI, and the probability of ancestral area associated with each node depicted in Figure 3 are in Supplementary Table S2 and Supplementary Figure S1.

## 4 Discussion

### 4.1 Phylogeny and biogeographic history

This study provides insights into the general processes shaping Ethiopian Highlands endemism, and further improves our understanding of the evolution of high species richness in tropical mountains. Our analysis of mitogenomes and nuclear SNPs of a comprehensive geographic and taxonomic sampling of the Ethiopian Highlands *Ptychadena*, suggests a complex history of diversification and dispersal from the east to the west and north of the GRV, that began in the middle-late Miocene. We propose that both the geological history of the Ethiopian Plateau uplift and the Great Rift Valley propagation (resulting in a complex topography), as well as environmental changes due to climatic oscillations, played a major role in the diversification of the species.

The mitogenomic phylogenetic inference obtained here is similar to the most recent studies using sequence data (Freilich et al., 2014; Smith et al., 2017a; Goutte et al., 2021), but with better node supports for all species relationships. The higher resolution of the mitogenomic data also allowed us to explore intraspecific phylogeographic structures. We acknowledge that our biogeographic reconstruction and times of divergence are only based on mitogenomic data, and could potentially lead to an overestimation of divergence time since the divergence of genes is always older than the divergence between species and populations (e.g., McCormack et al., 2011; Zheng et al., 2011). However, the times of divergence estimated here are similar to previous estimates using molecular clock analyses and both mitochondrial and nuclear sequences (Freilich et al., 2014), and coincide with important geological changes and climatic oscillations in this region. On the other hand, our estimates for the species diversification are older than those reported by Smith et al. (2017a). The different timings determined in the present study and by Smith et al. (2017a) may have resulted from different outgroup selection and node calibration. Smith et al. (2017a)) used the late Oligocene *Ptychadena* fossil (24.5 Mya, Blackburn et al., 2015) to calibrate the divergence between *Ptychadena* and *Micrixalus fuscus* (Boulenger, 1882), but this fossil is likely to be more recent than the split between *Ptychadena* and this outgroup (Feng et al., 2017; Dubois et al., 2021). This calibration may thus have resulted in an underestimation of divergence times across their tree. For example, Smith et al. (2017a) produced a divergence time between *Dyscophus guineti* (Grandidier, 1875) and *Ptychadena* around 30 Mya, while Feng et al. (2017), using a more comprehensive dataset, estimated this divergence to be at least 3 times older, around 100 Mya (Microhylidae vs. Afrobatrachia + Natatanura).

The most recent common ancestor of the Ethiopian Highlands *Ptychadena* was estimated to have occurred at around 11.7 Mya in our work. At this time, the Ethiopian plateau was lower (possibly by ∼1,000 m; Yemane et al., 1985; Adamson and Williams, 1987), and climatic and topographic conditions of the highland plateaus contrasted less sharply to those of the lowland in the rift than they do today. The complex topographic landscape prevailing in the Ethiopian Highlands results both from a series of regional tectonic uplift and volcanic events that occurred at ∼11.7, ∼6.5, and ∼4.5 Mya on the north-west and south-east plateaus (Sembroni et al., 2016; Xue et al., 2018), and from the development of the GRV. The ancestor of the *Ptychadena* clade probably colonized this changing environment and adapted to highlands in this period. Other species of endemic frogs also colonized the region during this period, for example, the monophyletic clade of the Highland *Leptopelis* (Reyes-Velasco et al., 2018b) and the genus *Paracassina* (Portik et al., 2019). Different lineages of Afroalpine vegetation prevailing until today in the highest elevations of the Ethiopian Highland plateaus also have colonized the region between 10 and 5 Mya (Kandziora et al., 2022), which support the presence of high-elevation environments during this period.

Most of the diversifications between sister species of Ethiopian Highlands *Ptychadena* species seem to have happened in the Pliocene. During this period, Earth’s climate gradually became drier and cooler (Robinson et al., 2008). The GRV was still under development, through the propagation of the Afar triple junction in the northern part and the Kenya Rift region in the south, and only completely opened in the central part of Ethiopia 3 Mya (Bonini et al., 2005). The co-occurrence of climatic and topographical changes probably allowed for the dispersal of species across the GRV at different times. Dispersal events were followed by periods of isolation, which were further promoted through the high abundance of microclimatic pockets across the topographically heterogeneous slopes and summits, leading to allopatric speciation within each plateau in the *P. neumanni* and *P. erlangeri* clades. The episodes of allopatric isolation of populations through recurrent expansion and contraction of suitable habitat due to climatic oscillations was suggested as main drivers of diversification for other anurans in Ethiopia (Evans et al., 2011; Freilich et al., 2016; Manthey et al., 2017; Reyes-Velasco et al., 2018b), as well as for other species, including mammals and birds (Bryja et al., 2018; Razgour et al., 2021; Manthey et al., 2022).

### 4.2 Phylogeography and population genetics

The repeated cycles of climatic oscillations and the resulting environmental changes were probably the major factor isolating lineages during the late Pliocene and Pleistocene, contributing to population structure in *P. cooperi*, *P. erlangeri* and *P. neumanni* throughout the last ∼3 My. These findings corroborate previous results by Smith et al. (2017a) and Reyes-Velasco et al. (2018a). Populations of these three species crossed the GRV during different climatic cycles. The relatively low nucleotide diversities found for all populations are consistent with a history of past bottlenecks driven by isolation and climatic oscillations.

The current environmental characteristics of the GRV (dry and hot) make it an important barrier to dispersal and gene flow for extant *Ptychadena* species adapted to the highlands. However, Freilich et al. (2016), using paleoclimate niche modeling, suggested that during the Holocene (last glacial maximum), environmental conditions might have allowed some species to expand to the valley and even cross it. This was probably the case for *P. neumanni* (*P. erlangeri* in Freilich et al., 2016) and *P. beka* (*P. cf. neumanni* 1 in Freilich et al., 2016). The absence of genetic structure at the nuclear and mitochondrial level among eastern and western populations of both species is consistent with these niche model results, supporting a very recent range expansion for these taxa. In addition, the population of *P. neumanni*-N1-West-East also shows signals of recent population expansion based on Tajima’s D. While *Ptychadena beka* presented negative values for Tajima’s D, these were not significant (*p* = 0.07, Table 1). Demographic analyses presented in Freilich et al. (2016) for both *P. neumanni* and *P. beka* also suggested moderate population expansion in the last 400,000 years.

Only four species and populations showed significant negative values for Tajima’s D (*P. amharensis, P. nana, P. robeensis, P. cooperi*-C2-Bale). This could be indicative of recent population expansion [e.g., following glaciation in the Ethiopian Highlands (e.g., Groos et al., 2021)] or selective sweeps due to new advantageous mutations (e.g., adaptation to the extreme environmental conditions in high elevations) reducing variation in linked neutral sites. For all other species Tajima’s D were not significant, but the low nucleotide diversities indicate rather low effective population size or past bottlenecks. Interestingly, Reyes-Velasco et al. (2018a) found signatures of reduction in population sizes for most species of the Ethiopian Highlands *Ptychadena* using nuclear data (RADseq). Contrasts between mitochondrial and nuclear makers should be further investigated in future studies.

### 4.3 Conservation implications

Our findings provide additional evidence that the Ethiopian Highlands represent important hotspots of biodiversity for amphibians (revealing multiples genetic lineages), as is also the case for other taxa (Gottelli et al., 2004; Largen and Spawls, 2010; Ceccarelli et al., 2014; Reyes-Velasco et al., 2018b; Kandziora et al., 2022). In face of the rapid human population growth and land use change in Ethiopia (Fashing et al., 2022), our findings also highlight the urgent need of IUCN conservation status assessment for the Ethiopian Highlands *Ptychadena*.

Most of the species we analyzed in this study have not been assessed for conservation status using IUCN criteria (*P. amharensis, P. beka, P. delphina, P. doro, P. goweri, P. levenorum, and P. robeensis*). For the other species, the last assessments were done in 2012 (IUCN, 2023). At that time, population structure and diversity of species in the clade were unknown, so distribution ranges considered (and possible threats) were based on polyphyletic lineages. The resolved taxonomy and novel genetic and geographical distribution data (present study; Reyes-Velasco et al., 2018a; Reyes-Velasco et al., 2021; Goutte et al., 2021) urge for IUCN (re-)assessments of the Ethiopian Highlands *Ptychadena* species, particularly in the light of contemporary climate change.

Currently, *Ptychadena nana* is listed as “Endangered” and *P. harenna* is listed as “Data Deficient”. Both species have restricted geographical distributions; *P. nana* is so far only known from the slopes of the Ahmar mountains north of the Shebelle River valley, while *P. harenna* is currently restricted to the Harenna forest on the southern slope of the Bale mountains. Particularly the status of *P. harenna* is in need of clarification and with the available knowledge an assessment has become possible. While the not assessed *Ptychadena goweri, P. levenorum* and *P. robeensis* also inhabit small distribution ranges (Figure 1) populations sizes, fragmentation and potential population decline require additional data collection which with the corrected nomenclature has now become possible. *Ptychadena erlangeri* is currently listed as “Near Threatened”, while *P. cooperi* and *P. neumanni* are considered “Least Concern”. Although these species have relatively large geographical ranges, we found population genetic structure for all of them. In addition, genetic data obtained more recently revealed confusion in the literature between *P. erlangeri* and *P. neumanni* (Reyes-Velasco et al., 2021). These findings highlight the need to incorporate the evolutionary history of the species on the conservation measures across the Ethiopian highlands.

## Data Availability

The data presented in the study are deposited in the https://www.ncbi.nlm.nih.gov/genbank repository, accession numbers OR211557-OR211559, OR223595-OR223753, OR224276-OR224319, OR224572-OR224604, OR228592-OR228648 and in the https://www.ncbi.nlm.nih.gov/sra/ repository, accession number PRJNA975347.
