# Supplementary figures and images for: Crossing the Rift valley: using complete mitogenomes to infer the diversification and biogeographic history of ethiopian highlands Ptychadena (anura: Ptychadenidae)
Source: Front Genet. 2023 Aug 3;14:1215715. doi: 10.3389/fgene.2023.1215715 (PMC10434514; doi:10.3389/fgene.2023.1215715)

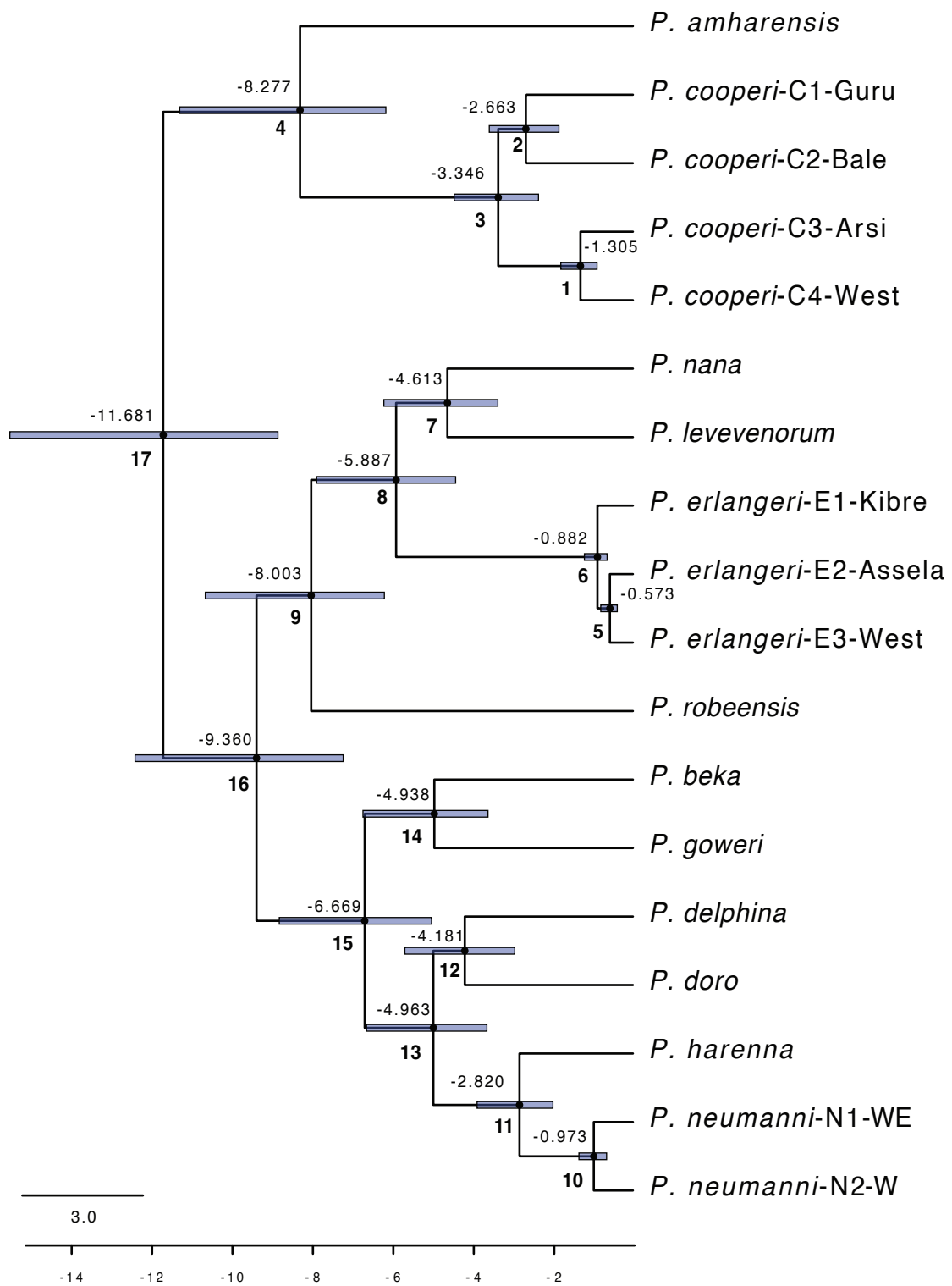

Supplement: Supplementary file 2 [file Image1.pdf]
